# Supplementary material for: Splice-Junction-Based Mapping of Alternative Isoforms in the Human Proteome
Source: Cell Rep. Author manuscript; Available in PMC 2020 Jan 15. (PMC6961840; doi:10.1016/j.celrep.2019.11.026)
Supplement: 2 [file NIHMS1546469-supplement-2.zip › DF1/PXD009737/TestisLysC/TestisLysC_40_SMARCC1_PGDVQGPGTAVGATGSGIAAAAAGLAVYRRKDGGPATK.pdf]

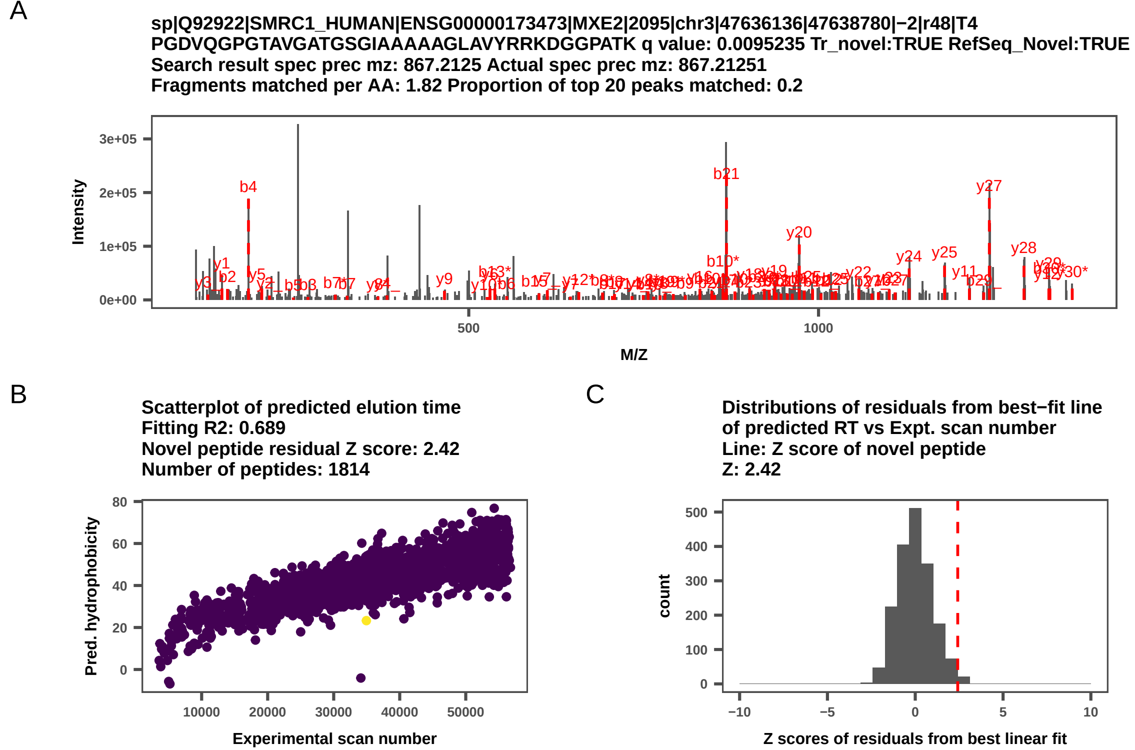
A
sp|Q92922|SMRC1_HUMAN|ENSG00000173473|MXE2|2095|chr3|47636136|47638780|−2|r48|T4
PGDVQGPGTAVGATGSGIAAAAAGLAVYRRKDGGPATK q value: 0.0095235 Tr_novel:TRUE RefSeq_Novel:TRUE
Search result spec prec mz: 867.2125 Actual spec prec mz: 867.21251
Fragments matched per AA: 1.82 Proportion of top 20 peaks matched: 0.2
Intensity
3e+05
2e+05
1e+05
0e+00
500
1000
M/Z
b4
b21
y27
y20
y24
y25
y28
y9
B
Scatterplot of predicted elution time
Fitting R2: 0.689
Novel peptide residual Z score: 2.42
Number of peptides: 1814
Pred. hydrophobicity
80
60
40
20
0
10000
20000
30000
40000
50000
Experimental scan number
C
Distributions of residuals from best−fit line
of predicted RT vs Expt. scan number
Line: Z score of novel peptide
Z: 2.42
count
500
400
300
200
100
0
−10
−5
0
5
10
Z scores of residuals from best linear fit
